# Humanized HLA-DR4 Mice Fed with the Protozoan Pathogen of Oysters *Perkinsus Marinus* (Dermo) Do Not Develop Noticeable Pathology but Elicit Systemic Immunity

**DOI:** 10.1371/journal.pone.0087435

**Published:** 2014-01-31

**Authors:** Wathsala Wijayalath, Sai Majji, Yuliya Kleschenko, Luis Pow-Sang, Teodor D. Brumeanu, Eileen Franke Villasante, Gerardo R. Vasta, José-Antonio Fernández-Robledo, Sofia Casares

**Affiliations:** 1 US Military Malaria Vaccine Program, Naval Medical Research Center/Walter Reed Army Institute of Research, Silver Spring, Maryland, United States of America; 2 Department of Medicine, Uniformed Services University of Health Sciences, Bethesda, Maryland, United States of America; 3 Department of Microbiology and Immunology, University of Maryland Baltimore School of Medicine, Baltimore, Maryland, United States of America; Université Libre de Bruxelles, Belgium

## Abstract

*Perkinsus marinus* (Phylum Perkinsozoa) is a marine protozoan parasite responsible for “Dermo” disease in oysters, which has caused extensive damage to the shellfish industry and estuarine environment. The infection prevalence has been estimated in some areas to be as high as 100%, often causing death of infected oysters within 1–2 years post-infection. Human consumption of the parasites *via* infected oysters is thus likely to occur, but to our knowledge the effect of oral consumption of *P. marinus* has not been investigated in humans or other mammals. To address the question we used humanized mice expressing HLA-DR4 molecules and lacking expression of mouse MHC-class II molecules (DR4.EA^0^) in such a way that CD4 T cell responses are solely restricted by the human HLA-DR4 molecule. The DR4.EA^0^ mice did not develop diarrhea or any detectable pathology in the gastrointestinal tract or lungs following single or repeated feedings with live *P. marinus* parasites. Furthermore, lymphocyte populations in the gut associated lymphoid tissue and spleen were unaltered in the parasite-fed mice ruling out local or systemic inflammation. Notably, naïve DR4.EA^0^ mice had antibodies (IgM and IgG) reacting against *P. marinus* parasites whereas parasite specific T cell responses were undetectable. Feeding with *P. marinus* boosted the antibody responses and stimulated specific cellular (IFNγ) immunity to the oyster parasite. Our data indicate the ability of *P. marinus* parasites to induce systemic immunity in DR4.EA^0^ mice without causing noticeable pathology, and support rationale grounds for using genetically engineered *P. marinus* as a new oral vaccine platform to induce systemic immunity against infectious agents.

## Introduction

*Perkinsus marinus* is a protozoan parasite that infects mollusk bivalves of both ecological and commercial interest. While its phylogenic placement has been subject of intense debate [1]–[6], now the genera *Perkinsus*, *Parvilucifera*, and *Rastrimonas* are included in the phylum *Perkinsozoa* [7], [8]. This phylum is considered to be the earliest group diverging from the lineage leading to dinoflagellates (responsible for harmful algal blooms), branching close to the node shared by dinoflagellates and apicomplexans [9]–[12]. Indeed, *Perkinsus* shares numerous gene products common to pathways and mechanisms identified in both dinoflagellate and apicomplexan [13], [14]. Identified in the early 1950’s on the Texas coast as the causative agent of “Dermo” disease in eastern oysters (*Crassostrea virginica*) [1], its distribution range includes both the Gulf of México and East and West coasts of North America [15]–[17]. Shellfish restocking and global warming may have contributed to the northward expansion of *P. marinus* infections on the Atlantic coast [15], [18], [19]. In the Chesapeake Bay (Maryland, Virginia, USA), *Perkinsus* outbreaks together with habitat loss and contamination have diminished the oyster populations to unprecedented low levels, with a significant detrimental impact in water quality and ecosystem integrity since oysters remove a considerable quantity of phytoplankton and silt from the water column [20], [21]. Six additional *Perkinsus* species affect mollusks worldwide [22]–[24]; indeed, Dermo disease is under surveillance by the World Organization for Animal Health (http://www.oie.int/).

The life cycle of *P. marinus* includes a free-living stage (zoospore) and a facultative intracellular stage (trophozoite). Trophozoites in the water column are taken up during filter-feeding by oysters, with the gut, mantle epithelium, gills, and labial palps suggested as primary portals of entry, although this has not yet been rigorously established [25]–[28]. Once inside the oyster, interaction between the galectin CvGal (C. *virginica* galectin) released by oyster hemocytes and surface ligands displayed on trophozoites leads to opsonization and phagocytosis of trophozoites [29]–[31]. Hemocytes are circulating phagocytic cells that intervene in the primary defense against pathogens, as well as in the digestion and transport of nutrients, and tissue and shell repair. Phagocytosed trophozoites remain inside a parasitophorous vacuole in the hemocytes where they resist oxidative killing and proliferate [31]–[35]. Migration of infected hemocytes through the oyster tissues leads to systemic infection and eventually death of the oyster within two years of infection [35]. Trophozoites from infected oysters are released into the water through pseudo-feces or from necrotic tissues or decaying dead oysters [36]. The released trophozoites can infect neighboring oysters to perpetuate the vegetative life cycle, or alternatively they may sporulate and after multiple rounds of division release hundreds of zoospores into the water column. Whether zoospores develop into trophozoites still remains uncertain [35]. The intensity and prevalence of *P. marinus* infections in oysters have seasonal trends and multi-year cycles determined by water temperature and salinity that have led to infection prevalence rates close to 100% in some areas [15], [37]. Human consumption of infected oysters is thus likely to occur frequently, nevertheless induction of gut pathology or oral immunity upon consumption of *P. marinus* infected oysters has not been investigated.

The Major Histocompatibility Complex (MHC, HLA in humans) molecules are critical for eliciting immune responses to microorganisms since their primary role is to present peptides for activation and differentiation of CD4 T cells [38]. Among the CD4 T cell subsets, CD4 T helper cells (Th1, Th2, Th17) are required to orchestrate cellular and humoral responses [39], while regulatory CD4^+^Foxp3^+^ T cells (Tregs) suppress cellular and/or humoral responses through direct cell-cell interactions or through the secretion of cytokines such as IL-10 and TGFβ [40], [41]. Since mouse MHC and human HLA class II molecules differ in their ability to present peptides, we used humanized mice expressing HLA-DR4 (B1*0401) molecules and lacking mouse MHC class II molecules (DR4.EA^0^) to determine whether *P. marinus-*derived antigenic peptides restricted by human HLA-DR4 molecules could drive immune responses upon oral consumption of the oyster parasite, and whether such responses might lead to pathology. HLA-DR4 (B1*0401) is one of the most common HLA-II alleles in humans [42]. Studies demonstrated that HLA-DR4 molecules expressed in transgenic DR4.EA^0^ mice present immunodominant epitopes of foreign-antigens and self-antigens to the same extent as they do in humans, and that the transgenic mice develop clinical and histological similarities of human autoimmune syndromes linked to HLA-DR4 [43], [44]. Our results indicated that naïve DR4.EA^0^ mice had pre-existent antibodies (IgM and IgG) that bind to the oyster parasite but lacked detectable cellular immunity. Feeding DR4.EA^0^ mice with live *P. marinus* resulted in specific cellular (IFNγ) responses and boosted humoral immunity to the oyster parasite, without inducing any noticeable pathology.

## Results

### *P. marinus* is Sensitive to Gastric pH and does not Shed from the Intestine

**Figure 1:**
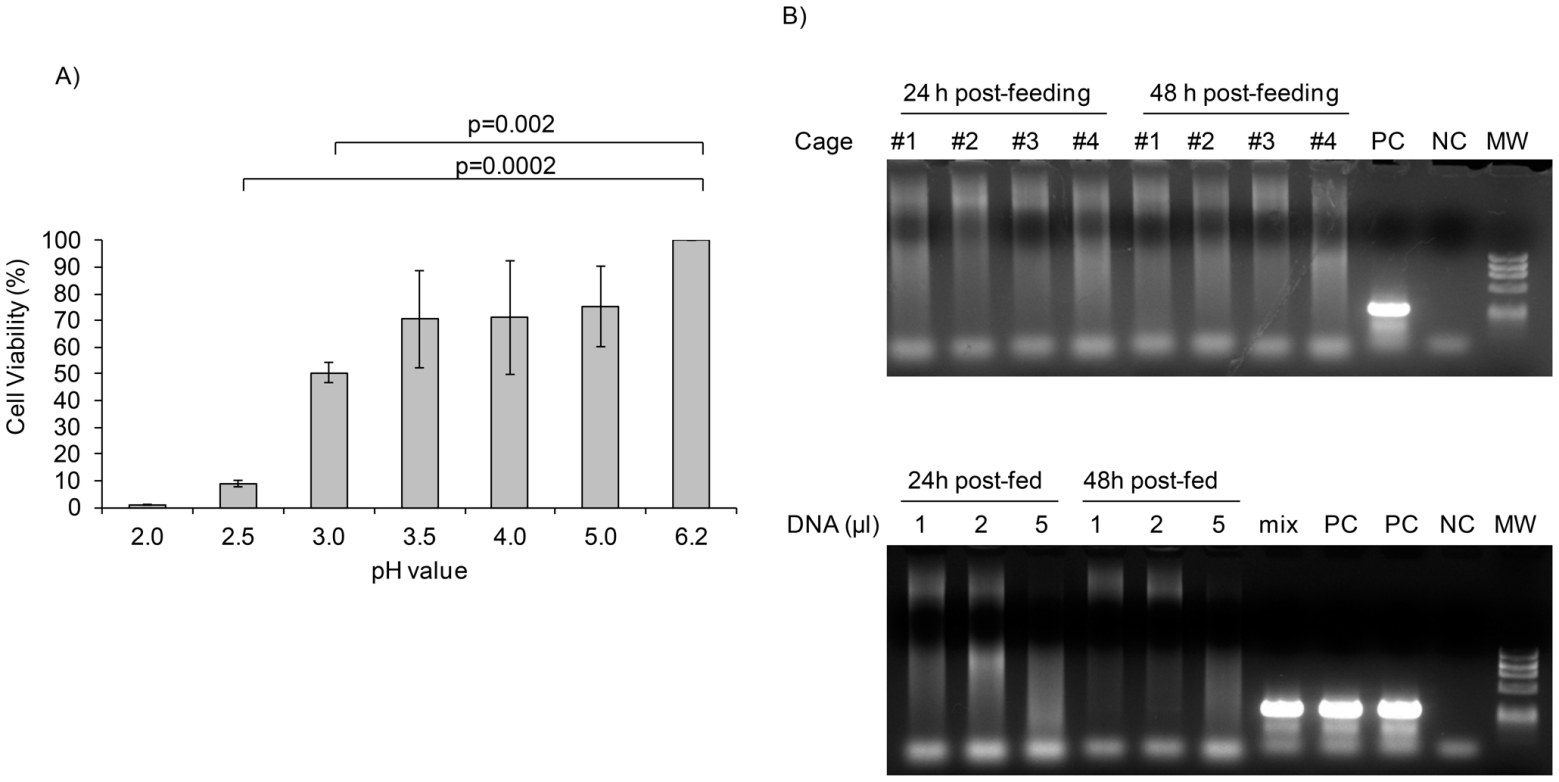
*P. marinus* is sensitive to gastric pH and does not shed from the intestine. **Panel A,** *P. marinus* parasites (7×10^5^ to 9×10^5^) were cultured for 10 minutes in media adjusted to pH values ranging from 2.0–6.2 and cell viability was measured by trypan blue exclusion. Data represent mean ± SD of two independent experiments. P values are indicated over the plots. **Panel B,** DR4.EA^0^ mice (n = 10) were fed with 10^5^ live *P. marinus* and set in 4 clean cages (2–3 mice per cage). Feces were collected at 24 h and 48 h post-feeding. DNA (10 ng) extracted from fecal samples was amplified with a pair of primers targeting NTS domain located between 5S and SSU rRNA genes from *P. marinus* (307 bp amplicon). Upper panel shows absence of parasite DNA in feces at 24 h and 48 h post-feeding. Lower panel shows that increasing concentration of fecal DNA (10 ng/µl) did not result in detectable PCR signal. Mixture of fecal DNA and purified parasite DNA (2∶1) resulted in positive PCR signal (lower panel) ruling out that potential inhibitory components in fecal material could have led to false negative results. PC, positive control; NC, negative control; MW, DNA molecular markers; Mix, Mixture of fecal DNA and purified *P. marinus* genomic DNA.

Gastric fluid contains hydrochloric acid (0.5%) and large quantities of potassium chloride and sodium chloride, thus providing an acidic pH (1.5–3.5) for proteases to exert catalytic activity [45] while preventing many microbial pathogens from reaching the gut [46]. We thus investigated the ability of *P. marinus* to survive at low pH values. As illustrated in Figure 1A, *P*. *marinus* cell viability gradually decreased when cultured for 10 minutes at pH values lower than 6.2 (marine pH 8.2–8.4) and only 50% of the parasites survived at pH 3.0. The parasites did not survive at pH values ≤2.0. Thus the results indicated that *P. marinus* is highly sensitive to the stomach acidic environment.

We next investigated whether orally administered *P. marinus* parasites could shed from the intestine of DR4.EA^0^ mice. For this, mice (n = 10) were fed with *P. marinus* (10^5^ per mouse in 100 µl) by gavage and feces were collected at 24 and 48 h post-feeding for analysis of the parasite DNA by PCR. As illustrated in Figure 1B, the PCR analysis of DNA extracted from the feces was negative. The fact that a PCR signal was detected upon *in vitro* mixture of fecal DNA with purified *P. marinus* DNA (Figure 1B, lower panel) ruled out that potential inhibitory components present in the fecal material could have led to false negative PCR results. The feces of DR4.EA^0^ mice examined at 24 and 48 h post-feeding with *P. marinus* were similar in size and morphology to those from control unfed mice (data not shown) ruling out that *P. marinus* induced diarrhea.

### Oral Administration of *P. marinus* does not Induce Gastrointestinal or Lung Pathology

**Figure 2:**
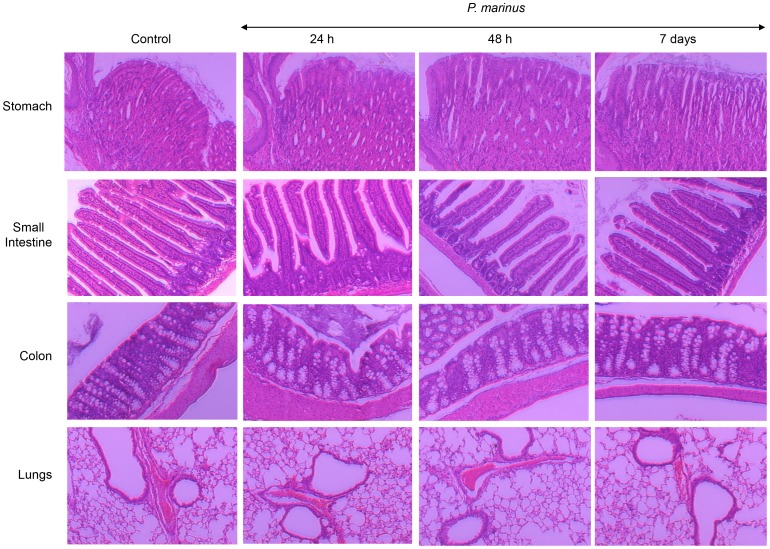
Oral administration of *P. marinus* does not induce gastrointestinal or lung pathology in DR4.EA^0^ mice. DR4.EA^0^ mice were fed by gavage with 10^5^ live *P. marinus* and euthanized at 24 h, 48 h, or 7 days post-feeding (n = 3 mice per time point) for histological examination. Unfed age-matched mice were used as controls (n = 3). Organs fixed in formaline were processed and stained with H&E. Representative images were acquired using light microscopy under 10× objective (100× total visual magnification).

Gastrointestinal infections and allergic reactions to food components are characterized by histological alterations in the gut architecture commonly due to disruption of the gastrointestinal epithelium and/or leukocyte infiltration [47], [48]. Gastrointestinal pathogens can also induce pulmonary pathology due to the close proximity of the esophagus and trachea. Thus we investigated whether *P. marinus* could induce histopathological alterations in the gastrointestinal tract or lungs of DR4.EA^0^ mice after gavage. For this, groups of mice (n = 3) were fed once with *P. marinus* and euthanized at 24 h, 48 h, or 7 days post-feeding and organs were used for histological examination. As illustrated in Figure 2, the stomach, small intestine, colon, and lungs of *P. marinus* fed mice were histologically unaltered and similar to that of control, unfed mice (n = 3). Same results were obtained in mice fed three times (at two-week apart) and analyzed 6 days post-third feeding (Figure S1), indicating the *P. marinus* neither induced gut nor lung pathology upon re-exposure. Increased levels of IFNγ in serum have been associated with infections and adverse events linked to vaccination [49], [50]. Mice fed once or twice with live *P. marinus* and examined at day 6 post-feeding, as well as control unfed mice, had undetectable levels of IFNγ in serum as measured by luminex (<1 pg/ml), which indicated that *P. marinus* did not induce infection or noticeable adverse events.

### Oral Administration of *P. marinus* does not Alter Lymphocyte Frequencies in the Gut

**Figure 3:**
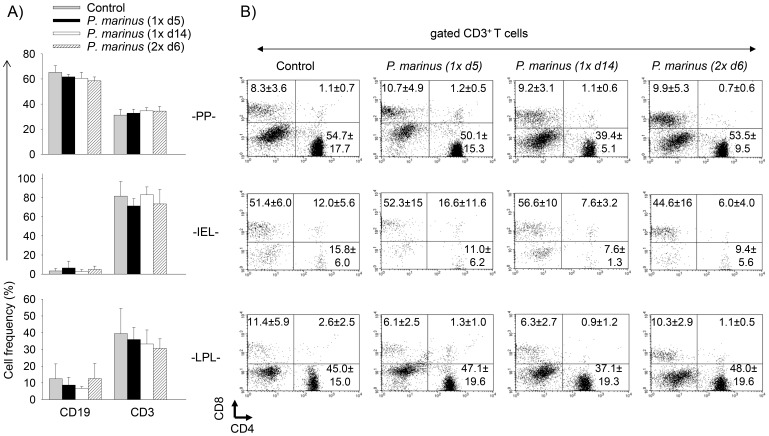
*P. marinus* does not alter the frequency of T and B cells in the gut. Groups of DR4.EA^0^ mice were fed with *P. marinus* once and examined at days 5 or 14 after the feeding (n = 6 mice per time point) or they were fed twice (at two week interval) and examined at day 6 after the second feeding (n = 6). Controls (n = 7) were unfed mice. Lymphocytes isolated from Peyer’s patches (PP), intraepithelial (IEL), and lamina propria (PPL) were stained with mouse CD3, CD4, CD8, and CD19 Abs and analyzed by FACS. **Panel A,** frequency of B (CD19^+^) and T (CD3^+^) cells in control and *P. marinus*-fed mice. **Panel B,** frequency of CD4^+^, CD8^+^, CD4^+^CD8^+^, and CD4^−^CD8^−^ T cell subsets among gated CD3^+^ T cells. Data represent mean ± SD of mice analyzed individually. There were no significant differences for the frequency of lymphocytes in the gut of control mice as compared to mice fed with *P. marinus* (p>0.05 determine by unpaired *t*-test).

The gastrointestinal immune system (Gut Associated Lymphoid Tissue, GALT) is organized into two major compartments: the “inductive” sites represented by Peyer’s patches (PP) and mesenteric lymph nodes, and the “effector” sites consisting of intraepithelial lymphocytes (IEL) and lamina propria lymphocytes (LPL) [51]. Thus we next investigated whether *P. marinus* could induce alterations in the GALT. For this, DR4.EA^0^ mice (n = 6) were fed once and examined at day 5 (1× d5) or day 14 (1× d14) after feeding, or they were fed twice (at two week intervals) and examined at day 6 after the last feeding (2× d6) for lymphocyte frequencies in the gut by FACS. Control mice were either left unfed (n = 7) or fed with media alone (n = 3). As illustrated in Figure 3A, there was no significant difference in the frequency of B (CD19^+^) and T (CD3^+^) cells in PP, IEL or LPL of *P. marinus*-fed mice as compared to control (unfed) mice. The frequency of T cell subsets (CD4^+^, CD8^+^, double positive CD4^+^CD8^+^, and double negative CD4^−^CD8^−^) was also similar between both groups of mice (Figure 3B). We also analyzed the frequency of CD4^+^Foxp3^+^ regulatory T cells (Tregs) at the induction and effector sites in the GALT, since the Tregs represent an important T cell compartment that is involved in induction of tolerance [51]. As shown in Table 1, the frequency of CD4^+^Foxp3^+^ Tregs in PP, IEL and LPL was also similar between *P. marinus*-fed and control mice. All together, these data indicated that repeated exposure to *P. marinus* did not alter the frequencies of T and B cells in the GALT of DR4.EA^0^ mice.

**Table 1:** Frequency of regulatory CD4^+^Fop3^+^ T cells (Tregs) in the gut.

	Control		*P. marinus*-fed		
	Unfed	Media-fed	1× day 5	1× day 14	2× day 6
**PP**	1.6±0.9	1.2±0.2	1.4±0.5	1.5±0.5	1.2±0.2
**IEL**	1.0±0.7	0.4±0.1	1.0±0.6	1.6±0.2	0.8±0.2
**LPL**	2.2±1.1	1.6±0.1	1.0±0.7	2.2±1.8	1.4±1.1

PP, Peyer’s patches (PP); IEL, intraepithelial lymphocytes, LPL lamina propria lymphocytes. There were no significant differences between control (unfed) and *P. marinus*-fed mice (p>0.05 unpaired two-sample *t*-test).

### Oral Administration of *P. marinus* Stimulates Specific Immune Responses

**Figure 4:**
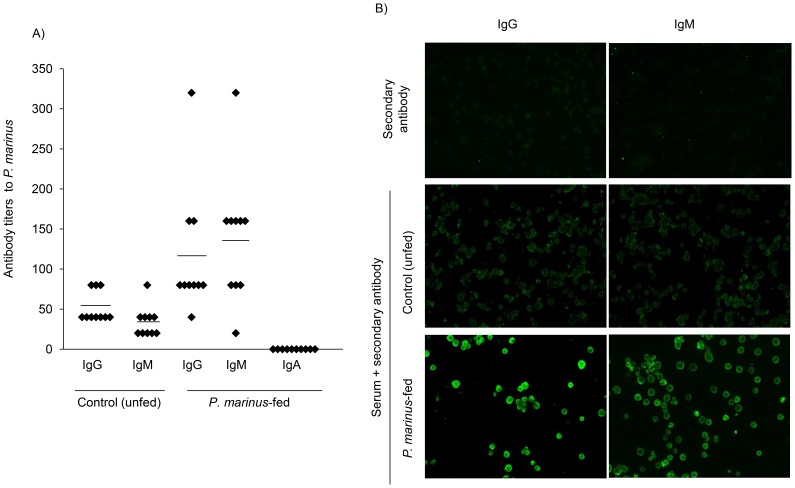
Humanized mice elicit IgG and IgM responses following oral feeding with *P. marinus*. **Panel A,** IFA antibody titers in naïve (unfed) mice and mice fed with *P. marinus* (twice at two-week interval) measured at two weeks post-second feeding. Data represent titers in ten mice analyzed individually. **Panel B** shows representative IFAs.

**Figure 5:**
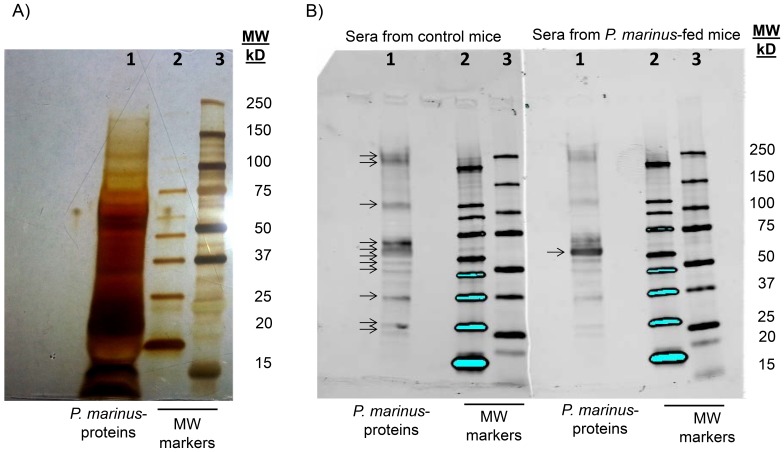
Western blot analysis of IgG antibodies to *P. marinus*. **Panel A,** *P. marinus* proteins extracted by thawing-freezing/ultrasonication as described in material and methods were separated in 4–15% SDS-PAGE gradient gel and silver-stained (lane 1); shown are the MW markers in lanes 2 (MagicMark) and 3 (Odyssey); **Panel B** show the same *P. marinus* protein sample after probing with sera from naïve mice (left) and from *P. marinus*-fed mice (right). The arrows indicate the major *P. marinus* protein bands recognized by IgG serum antibodies from naïve mice (arrows in panel B left, lane 1) and the most abundant *P. marinus* protein of approximately 60 kDa recognized by IgG serum antibodies from mice fed with *P. marinus* (arrow in panel B right, lane 1).

We next investigated whether feeding of DR4.EA^0^ mice with *P. marinus* could induce immune responses to the oyster parasite. For this DR4.EA^0^ mice were fed with *P. marinus* twice at two week-interval and examined two weeks later for specific antibodies by immunofluorescence (IFA) using slides coated with *P. marinus*. As control, we used sera from naïve (unfed) DR4.EA^0^ mice and naïve wild type C57BL/6 mice. Interestingly enough, all control (unfed) DR4.EA^0^ mice examined (n = 10) had pre-existent IgM and IgG, but not IgA, antibodies cross-reactive to the oyster parasite (Figure 4A & B) and the same was true for naïve, wild type C57BL/6 mice (n = 3, antibody titers IgM 80+/−69; IgG 53+/−23). This indicated that the presence of antibodies reactive to *P. marinus* in naïve mice is unrelated to their MHC genotype. Feeding DR4.AE^0^ mice with *P. marinus* significantly increased the titers of specific IgM (p = 0.003) and IgG antibodies (p = 0.035) but the fed mice failed to elicit specific IgA antibodies (Figure 4A). To further investigate the antibody responses in control and *P. marinus*-fed mice, we carried out Western blot analysis using *P. marinus* protein extracts under denaturing/reducing conditions. Figure 5A shows *P. marinus* protein extracts silver-stained. As illustrated in Figure 5B, the serum IgG antibodies from naïve as well as *P. marinus-*fed DR4.EA^0^ mice recognized eleven major *P. marinus* proteins, though the largest amount of IgG antibodies from mice fed with *P. marinus* recognized a 60 kDa protein component (Figure 5B, right panel). In aggregate, these results demonstrated that naïve mice have pre-existent IgG antibodies to *P. marinus*, and that feeding mice with live *P. marinus* boosted IgG humoral responses against a single *P. marinus* protein component of 60 kDa.

**Figure 6:**
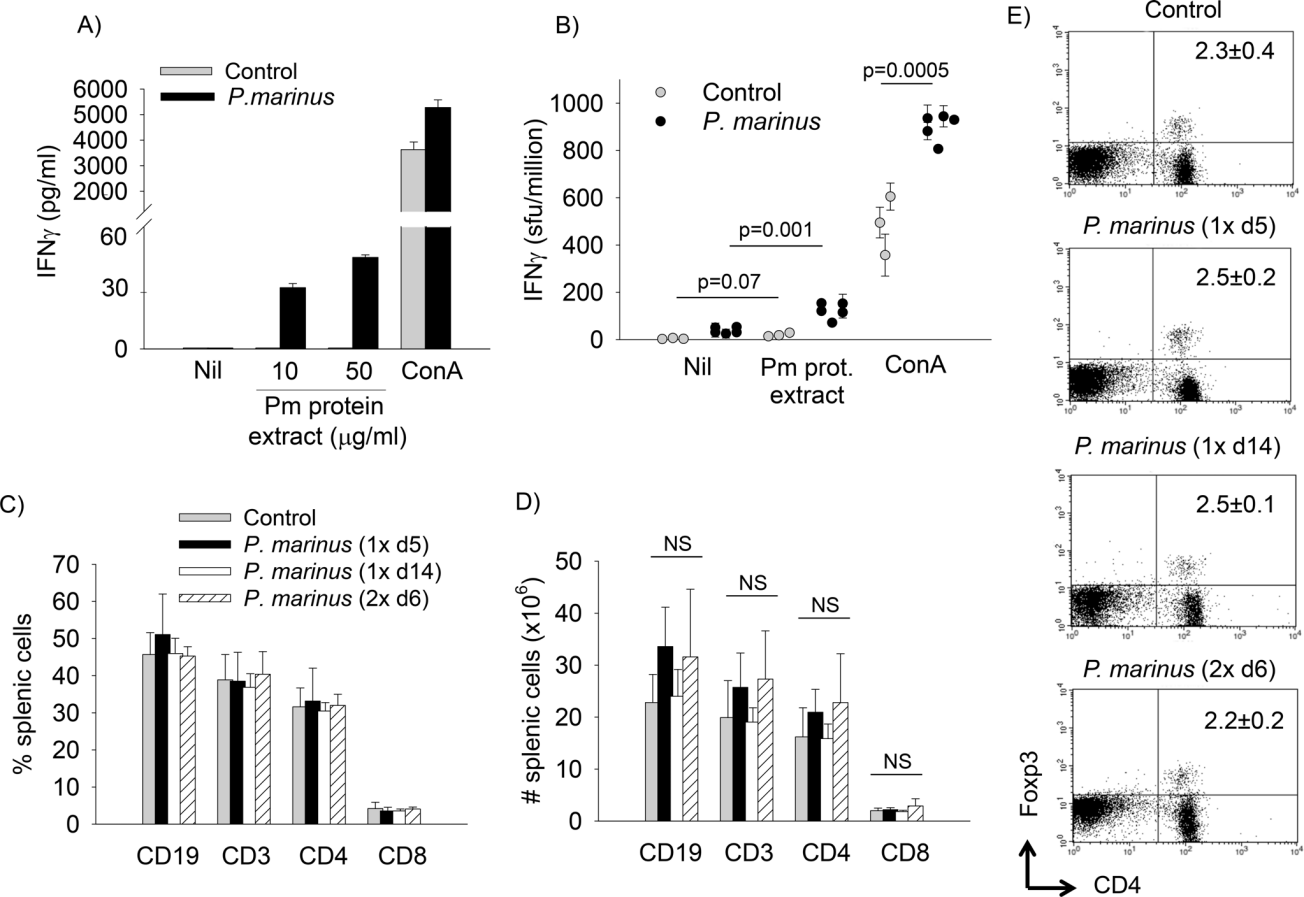
DR4.EA^0^ mice fed with *P. marinus* elicit cellular immunity. **Panel A,** groups DR4.EA^0^ mice were fed with *P. marinus* twice at two-week interval and euthanized six days later. Controls were unfed mice. Spleen cells were stimulated with ConA (2 days) or *P. marinus* protein extract (4 days) and the levels of IFNγ in cell culture supernatants were measured by ELISA. Data represent mean ± SD of triplicate samples from three pooled spleens. **Panel B,** in an independent second experiment mice were fed with *P. marinus* as in panel A or unfed (control). Splenic cells were stimulated in triplicated samples (2 days) with *P. marinus* protein extracts or ConA and analyzed by ELISPOT. Control cultures were non-stimulated. Data represent mean spot forming units (sfu)/10^6^ cells ± SD of triplicated samples from control (n = 3) and *P. marinus*-fed mice (n = 5) analyzed individually. T cells from control mice did not produce IFNγ upon stimulation with *P. marinus* protein extracts (p = 0.07, paired *t*-test) while T cells from *P. marinus*-fed responded to stimulation (p = 0.001, paired *t*-test). The T cell response to polyclonal stimulation with ConA was significantly higher in spleens of *P. marinus*-fed mice as compared to control mice (p = 0.0005, unpaired t-test). **Panel C&D,** Frequencies and numbers of B cells (CD19^+^), T cells (CD3^+^), CD3^+^CD4^+^ and CD3^+^CD8^+^ T cell subsets in spleens of mice fed once with *P. marinus* and examined at day 5 (1× d5) or day 14 (1× d14) post-feeding, mice fed twice with *P. marinus* (at two-week interval) and examined at day 6 post-feeding (2× d6) or control (unfed) mice. **Panel E,** Frequency of regulatory Foxp3^+^ T cells in spleens on *P. marinus*-fed and control mice. Data in panels C–E represent mean ± SD of six mice fed with *P. marinus* and seven control (unfed) mice analyzed individually. NS, not significant (p>0.05) determined by unpaired *t*-test.

Naïve DR4.EA^0^ mice did not have detectable T cell responses to *P. marinus* in spleen as measured by ELISA (Figure 6A) and ELISPOT (Figure 6B), suggesting that the pre-existent antibodies reactive to *P. marinus* could be T-cell independent. However, splenic T cells from mice fed with live *P. marinus* produced IFNγ (Figure 6A&B), which indicated that the *P. marinus*-fed DR4.EA^0^ mice elicited cellular immunity to the oyster parasite. Upon polyclonal T cell stimulation with ConA, the splenic T cells from *P. marinus*-fed mice secreted higher levels of IFN-γ than those from control (unfed) mice (Figure 6A & B), despite a similar frequency (Figure 6C) and numbers (Figure 6D) of T cells in the spleens. This can be explained by the presence of a previously activated pool of *P-marinus* reactive T cells in the spleen, as pre-activated T cells are known to secrete higher levels of IFNγ upon re-stimulation than naïve T cells [52]. The frequency of CD4^+^Foxp3^+^ Tregs was also similar in the spleens of fed and control (unfed) mice (Figure 6E), which indicated that *P. marinus* did not alter the splenic Treg compartment.

## Discussion

Most diseases outbreaks linked to eaten raw oysters originated from oysters concentrating pathogens (bacteria and viruses) present in the water column and derived from runoffs and sewage [53]–[57]. However, to our knowledge, the effect of consumption of raw oysters infected with *P. marinus* has not been investigated in humans or other mammals. Our results in humanized mice expressing human HLA class II (DR4) molecules and lacking expression of mouse MHC-II molecules indicated that consumption of *P. marinus* parasites does not induce pathology as shown by (i) histological analysis of the gastrointestinal tract and lungs revealing unaltered tissue and lack of inflammation, (ii) presence of normally shaped feces ruling out diarrheal symptoms, and (iii) normal IFNγ serum levels and counts of lymphocyte populations in the gut and spleen ruling out local or systemic infection. The results demonstrating that *P. marinus* does not induce pathology in humanized mice should not to be interpreted as the lack of potential pathological reactions in humans consuming *P. marinus*-infected oysters. However it is noteworthy that in some geographical areas such as the Chesapeake Bay (USA) the prevalence of *P. marinus*-infected oysters is very high [15], [58], but no significant epidemiological reports have been associated with oyster consumption (http://edis.ifas.ufl.edu/fa178).

Interestingly enough, naïve DR4.EA^0^ mice as well as naïve wild type C57BL/6 mice had IgM and IgG antibodies cross-reacting with whole *P. marinus* parasites. Since rodents are fed commercial food that contains fish meal (obtained from seafood and small marine fish that is cooked, pressed, dried, and/or grinded) it is difficult to elucidate whether the antibodies specific for *P. marinus* in mice are “natural antibodies” (meaning antibodies produced in the absence of antigen stimulation) or whether these antibodies could have been elicited upon feeding with commercial food containing marine products. Naïve mice however did not have detectable cellular responses to *P. marinus* in spleens suggesting that the pre-existent humoral immunity to *P. marinus* in naïve mice could be T-cell independent. Upon feeding with live *P. marinus,* DR4.EA^0^ mice had detectable cellular responses in spleen characterized by IFNγ secretion, and the titers of specific IgM and IgG antibodies significantly increased, indicating live *P. marinus* stimulated both humoral and cellular responses to the oyster parasite.

Orally delivered antigens are processed and presented to the digestive tract immune system, known as GALT. While most food antigens are presented in the GALT in a non-immunogenic form that induces tolerance and prevents allergic reactions, other food antigens can induce local immune responses in the gastrointestinal mucosa (characterized by IgA isotype) or systemic immunity (characterized by IgG isotype) [51]. The underlying mechanisms for selective induction of tolerance versus immunity to orally administered antigens remain largely unknown, though some evidence indicates that the level of protein degradation in the gastrointestinal tract may play an important role. Thus, proteins highly degraded in the upper gastrointestinal tract are likely to induce tolerance, while proteins resistant to degradation may retain immunogenicity and induce local immune responses in the gut, or systemic immunity if the antigens can transvasate through the gut [51]. This concept is in part supported by the fact that microorganisms able to replicate in the gut or particulate vaccine preparations designed to protect entrapped antigens from degradation can induce local or systemic immune responses [59]. Mice fed with *P. marinus* failed to elicit specific IgA antibodies, which indicated that live *P. marinus* induced systemic but no local immunity. The fact that mice fed with *P. marinus* developed IgG antibodies preferentially to a *P. marinus* protein component of approximately 60 kDa strongly suggests an increased immunogenicity of this protein(s) rather than high abundance among other proteins. This is because other abundant protein component of 25–30 kDa in the *P. marinus* protein extract were unable to induce IgG specific antibodies above the level observed in naïve mice. The *Perkinsus marinus* genome encodes for 23,654 predicted proteins, from which 263 proteins are within the 60 kDa range (roughly 545 amino acids in length) (http://img.jgi.doe.gov/cgi-bin/w/main.cgi?section=TaxonDetail&page=taxonDetail&taxon_oid=649328904). The predicted function for these proteins is very broad with the larger number belonging to the group of chaperonins and protein kinases, though more than 30% of these proteins are of unknown function. Ongoing studies are focused on the identification of the immunogenic *P. marinus* protein(s) in humanized mice and whether such protein(s) can be genetically engineered to express immunodominant B and T cell epitopes of relevant infectious disease agents.

Current licensed vaccines delivered by the oral route are aimed at inducing local (gut) immunity in order to prevent disease contracted through the gut mucosa such as polio, cholera, typhoid, rotavirus, and adenovirus. In contrast there has been little progress for development of oral vaccines able to induce systemic immunity to pathogens that do not enter through the oral route [60]. Unlike vaccines delivered by injection, oral vaccines make vaccination of large populations less expensive and importantly safer, particularly in developing countries whether the reuse of needles has been linked to vaccination-related infections [61]. The ability of *P. marinus* to elicit systemic immunity, characterized by IgG antibodies and IFNγ secretion provides a rationale for the use of genetically-engineered *P. marinus* parasites expressing antigenic proteins/subunits of infectious agents to induce protective immunity through the oral route. This is especially relevant for diseases caused by apicomplexan parasites for which there are not effective vaccines [62]. Based on the large number of genes and pathways shared by *Perkinsus* and the apicomplexan [13], [14], and the availability of a transfection system [63], this approach has the potential to become an alternative for delivering vaccines against the apicomplexan parasites.

## Materials and Methods

### Ethics Statement

All animal procedures reported herein were conducted under protocols approved by the Institutional Animal Care and Use Committees at Walter Reed Army Institute of Research/Naval Medical Research Center (permit #11-IDD-31) and Uniformed Services University of Health Sciences (permit #G187A1) in compliance with the Animal Welfare Act and in accordance with the principles set forth in the ‘‘Guide for the Care and Use of Laboratory Animals,’’ Institute of Laboratory Animals Resources, National Research Council, National Academy Press, 1996.

### Mice

The DR4.EA^0^ (C57BL/6) mice express transgenically (HLA-DR*0401α1β1/I-E^d^α2β2) molecules under the I-E^d^ promoter and at the same time lack expression of mouse MHC-II molecules (AbbKO mutation, EA^0^) [44]. The chimeric HLA-DR*0401/I-E^d^ molecules allow binding of peptides to the HLA-DR*0401 groove while preserving interaction of mouse CD4 molecules to the I-E^d^ domain. The DR4.EA^0^ mice were purchased from Taconic (Hudson, NY) and bred at WRAIR/NMRC Animal Facility.

### Parasites

*Perkinsus marinus* CB5D4 (ATCC PRA-240 strain; http://www.atcc.org/) [63], [64] were cultured at room temperature in DME: Ham's F12 (ratio 1∶2) and supplemented with 5% fetal bovine serum (FBS) as previously described [65]. To determine the effect of pH on *P. marinus* survival, cultured parasites (7×10^5^–9×10^5^) were suspended in 100 µl of complete media previously adjusted to pH ranging from 2.0 to 6.2. Cell viability upon culture for 10 minutes at room temperature was measured by trypan blue exclusion.

### Oral Immunization

Oral feeding (gavage) was carried out under sterile conditions, using disposable animal feeding needles (Fisher Scientific, Pittsburgh, PA) fitted to 1 mL syringes. Each oral inoculum consisted of 10^5^ live *P. marinus* parasites suspended in 100 µl of complete culture medium.

### Histology

The gastrointestinal tract was rolled into a “Swiss roll” on a solid piece of paper prior to fixation in 10% formalin and the paper support was removed following 24 h fixation. Lungs were infused with 10% formalin prior to harvesting**.** The formalin fixed samples were embedded in paraffin blocks and histological sections were stained with hematoxylin-eosin (H&E) (HistoServ, Gaithersburg, MD).

### Fecal PCR Testing

Mice fed with *P. marinus* (n = 10) were set in four clean cages (2 to 3 mice per cage) to collect feces dropped at 24 h post-feeding and then set again in clean cages to collect feces dropped at 48 h post-feeding. DNA was extracted from fecal droppings using ExtractMaster™ Fecal DNA extraction kit (Epicentre Biotechnologies, Madison, WI). A pair of primers was used to amplify a 307 bp region in NTS domain located between 5S and SSU rRNA genes of *P. marinus* [66]. Primers and PCR cycling conditions were as described [67] with the following modifications. PCR amplification was carried out in 20 µL reaction volume containing DNA (10–50 ng), 1.0 unit of *Taq* polymerase (Promega, Madison, WI), 0.375 µM each of forward and reverse oligonucleotide primers and 1 mM deoxynucleotide triphosphates (dNTPs) mix (Roche Diagnostics, Indianapolis, IN) in reaction buffer containing 2 mM MgCl2 (InVitrogen, Carlsbad, CA). PCR amplified products were analyzed on 1.5% agarose gel stained with Ethidium Bromide.

### Isolation of Lymphocytes

Small intestine was isolated and dissected into ice cold RPMI supplemented with 10% FBS and gentamycin, and mesentery, residual fat, and other connective tissues were removed. Peyer’s patches (PP) were identified along the intestine using a light source and excised in ice cold RPMI/FBS/gentamycin. PP lymphocytes were isolated by gently forcing PP through a 70 µm cell strainer. PP lymphocytes were pelleted at 1,200 rpm for 10 min (+4°C) and re-suspended in RPMI/FBS/gentamycin. Isolation of intraepithelial lymphocytes (IEL) and lamina propria lymphocytes (LPL) was carried out as described [68]. Splenocytes were isolated as described previously [69].

### Immunofluorescence Assay (IFA)

Teflon printed 12-well slides (Electron Microscopy Sciences, Hatfield, PA) were coated with 3×10^4^ *P. marinus* parasites in 1× PBS/1%BSA/well, air-dried and stored at −80°C until use. Slides were thawed for 30 min at room temperature and then blocked with 1xPBS/1% BSA for 30 min at 37°C. Serum samples at various dilutions (two-fold dilution series starting at 1∶20) were added to the parasite-coated wells and incubated for 1 h at 37°C. Slides were washed three times with 1xPBS, incubated with FITC-labeled F (ab’)_2_ goat anti-mouse IgM, IgG, or IgA (Southern Biotechnologies, Birmingham, AL) at a 1∶40 dilution in 1xPBS/0.1% Evans blue for 30 minutes at 37°C. Slides were washed, air dried, and mounted with VECTASHIELD-DAPI (H-1200, Vector laboratories, Burlingame, CA).

### Cytokine Secretion

To prepare protein extracts for *in vitro* stimulation, *P. marinus* parasite cultures (10^8^) were washed twice in 1xPBS and suspended in 3 mL of mini protease inhibitor (Roche Diagnostics)**.** The parasite suspension was freeze-thawed three times using liquid nitrogen and boiling water, followed by sonication at 75% amplitude, with 20 sec on, 10 sec off for 3 min. Cell lysates were centrifuged at 10,000 rpm/4°C for 15–20 min and protein concentration was measured by Biuret. Splenocytes (5×10^5^) were stimulated with ConA (2.5 µg/mL, Sigma-Aldrich, St. Louis, MO) for 48 h or with 10 µg/mL or 50 µg/mL of *P. marinus* protein extract for 4 days. Control cultures were left unstimulated. Mouse IFNγ responses in cell culture supernatants were measured by ELISA (InVitrogen). The levels of mouse IFNγ in serum were measured by Luminex (Invitrogen).

### Interferon-gamma Enzyme Linked Immunospot (ELISPOT)

ELISPOT assays were performed using Mouse IFNγ ELISPOT kits (BD Biosciences, San Jose, CA) following the manufacturer’s instructions. Briefly, splenic cells (4×10^5^) were cultured in plates pre-coated with anti-mouse IFNγ and stimulated for 2 days with *P. marinus* proteins extracts (50 µg/ml), ConA (2.5 µg/ml) or left unstimulated. Biotinylated anti-mouse IFNγ antibody, Streptavidin –HRP, and AEC substrate were used to capture and visualize the IFNγ spots. Image analysis and spot enumeration was performed using an AID ELISPOT reader (Advanced Imaging Devices Gmbh, Strasberg, Germany).

### FACS Analysis

Splenocytes, PP lymphocytes, IELs, and LPLs were surface stained with anti-mouse CD3, CD4, CD8, and CD19 antibodies (BD Biosciences). For staining of Tregs, cells were cell-surface stained with anti-mouse CD3, CD4, and CD25 antibodies (BD Biosciences) and intracellularly stained with rat anti-mouse Foxp3 (eBiosciences, San Diego, CA). Cells were gated and analyzed on the mononuclear FSC/SSC scatter.

### Western Blot

*P. marinus* protein extracts obtained as above were suspended in Laemmli loading buffer (Bio-Rad, Hercules, CA) containing 2-mercaptoethanol (5%). Protein molecular weight markers used were MagicMark (InVitrogen) and Odyssey (LI-COR, Lincoln, NE). Samples were boiled for 10 minutes and separated in 4–15% SDS-PAGE gels (Bio-Rad). Gels were electotransferred on Odyssey Nitrocellulose Membranes (LI-COR) blocked for 1 h with Odyssey Blocking buffer (LI-COR), probed overnight with sera from control mice or mice fed with *P. marinus*, and revealed with goat anti-mouse IgG Alexa-fluor 680 (ThermoFisher Scientific, Rockford, IL). Membranes were visualized using a LI-COR Aerius Automated Infrared Imaging system.

### Statistical Analysis

Independent-samples (unpaired) *t*-test was used for comparison of lymphocyte frequencies between groups of mice. Paired two-sample *t*-test was used for comparison of ELISPOT responses prior and post-stimulation.

## Supporting Information

Figure S1 **Repeated feedings with** ***P. marinus*** **does not induce gut pathology.** Formalin-fixed, hematoxylin/eosin stained sections (10x) of stomach, small intestine, colon, and lungs. DR4.EA^0^ mice (n = 3) were fed by gavage with 10^5^ live *P. marinus* three times at two-week apart and euthanized on day 6 post-third feeding for histological examination. Unfed age-matched mice were used as controls.
(TIF)
