# Supplementary figures and images for: Humanized HLA-DR4 Mice Fed with the Protozoan Pathogen of Oysters Perkinsus Marinus (Dermo) Do Not Develop Noticeable Pathology but Elicit Systemic Immunity
Source: PLoS One. 2014 Jan 31;9(1):e87435. doi: 10.1371/journal.pone.0087435 (PMC3909113; doi:10.1371/journal.pone.0087435)

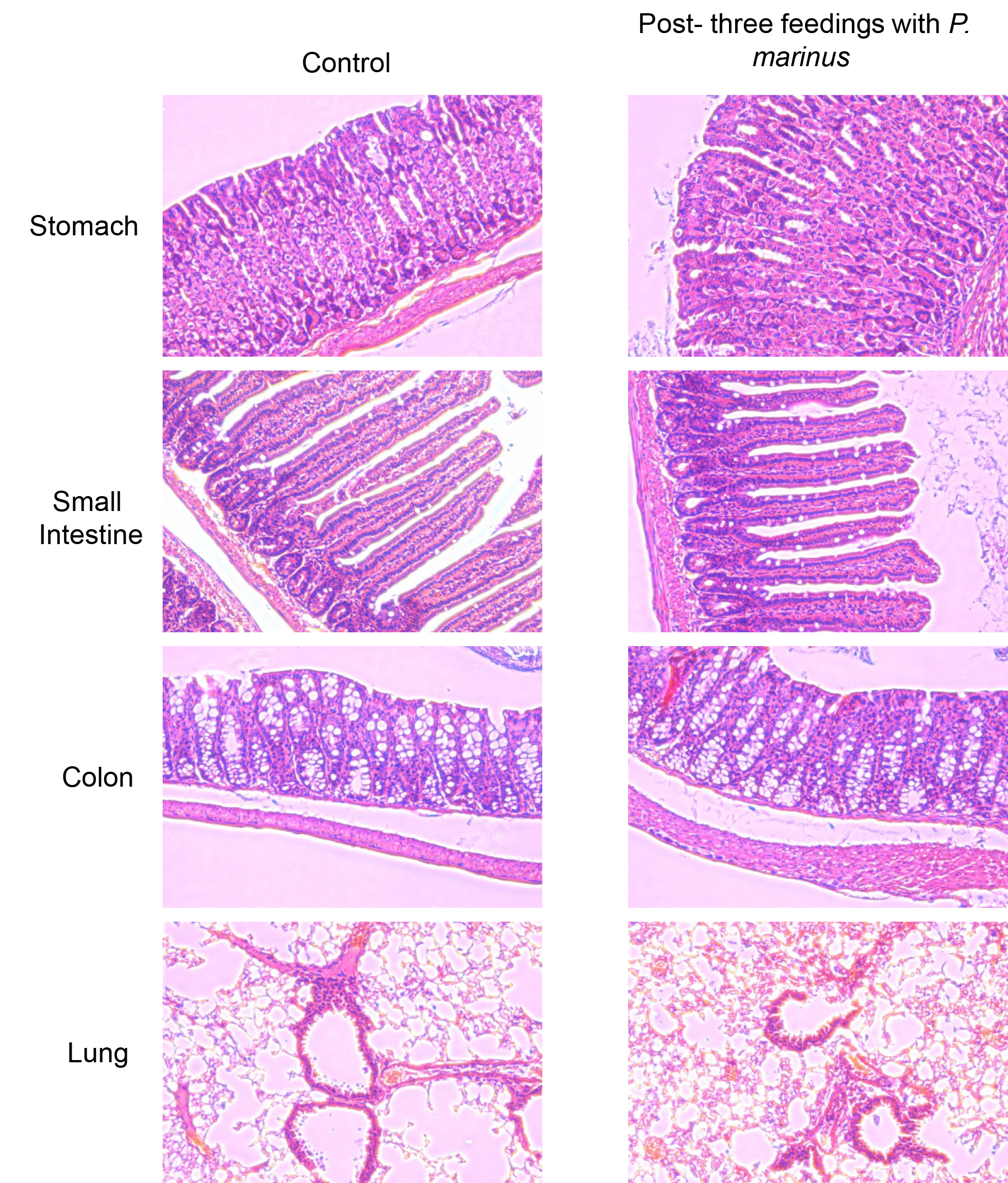

Supplement: Figure S1 — Repeated feedings with P. marinus does not induce gut pathology. Formalin-fixed, hematoxylin/eosin stained sections (10x) of stomach, small intestine, colon, and lungs. DR4.EA0 mice (n = 3) were fed by gavage with 105 live P. marinus three times at two-week apart and euthanized on day 6 post-third feeding for histological examination. Unfed age-matched mice were used as controls. (TIF) [file pone.0087435.s001.tif]
